# Four centuries on from Bacon: progress in building health research systems to improve health systems?

**DOI:** 10.1186/1478-4505-12-56

**Published:** 2014-09-23

**Authors:** Stephen R Hanney, Miguel A González-Block

**Affiliations:** Health Economics Research Group, Brunel University London, Uxbridge, UK; Universidad Anáhuac, Mexico City, Mexico

**Keywords:** Collaborative approach, Health research system, Health systems, Payback framework, Research agenda setting, Research capacity, Research impacts, Research to policy, Value of medical research, World Health Report

## Abstract

In 1627, Francis Bacon’s *New Atlantis* described a utopian society in which an embryonic research system contributed to meeting the needs of the society. In this editorial, we use some of the aspirations described in *New Atlantis* to provide a context within which to consider recent progress in building health research systems to improve health systems and population health. In particular, we reflect on efforts to build research capacity, link research to policy, identify the wider impacts made by the science, and generally build fully functioning research systems to address the needs identified.

In 2014, *Health Research Policy and Systems* has continued to publish one-off papers and article collections covering a range of these issues in both high income countries and low- and middle-income countries. Analysis of these contributions, in the context of some earlier ones, is brought together to identify achievements, challenges and possible ways forward. We show how 2014 is likely to be a pivotal year in the development of ways to assess the impact of health research on policies, practice, health systems, population health, and economic benefits.

We demonstrate how the increasing focus on health research systems will contribute to realising the hopes expressed in the World Health Report, 2013, namely that all nations would take a systematic approach to evaluating the outputs and applications resulting from their research investment.

## Editorial

In 1627, Francis Bacon’s *New Atlantis*, an unfinished book published a year after his death, described a utopian society that included an embryonic research system which contributed to meeting society’s needs, although it did so on its own terms [1]. Some of the aspirations highlighted in *New Atlantis* are still extant and provide a useful context within which this editorial explores recent progress in building health research systems to improve health systems and population health. In particular, we reflect on progress, and consider prospects for efforts to build research capacity, link research to policy, identify the wider impacts made by the science, and, generally, build fully functioning research systems addressing the needs identified.

In *New Atlantis*, the narrator of the tale describes what he was told by the head of the fictional society’s scientific college. The numbers of people involved were not large, but worked together and in a diversity of roles set in a framework that prefigured features of a modern research system. Some of the team of scientists collected experiments by travelling to other countries, some went through books to collect experiments, some collected the experiments of *‘all mechanical arts’*, i.e., practical fields, and some ‘*try new experiments, such as themselves think good*.’ It seems fair to ask whether Bacon also possibly foresaw the role for systematic reviewers in the description given of a fifth group in the team who ‘*draw the experiments of the former four into titles and tables, to give the better light for the drawing of observations and axioms out of them. These we call compilers.*’ [1].

The use of research was important in the utopian society; there was another group who would contribute by: ‘*looking into the experiments of their fellows, and cast about how to draw out of them things of use and practice for man’s life and knowledge*’. And, as would be recommended in any properly functioning health research system, yet another team of scientists would build on the findings to inform further research, but in this case would do so only after a team effort to identify the best way forward: ‘*Then after divers meetings and consults of our whole number, to consider the former labours and collections, we have three that take care out of them to direct new experiments, of a higher light, more penetrating into nature than the former. These we call lamps.*’ [1].

### Building health research capacity

Nearly 400 years on, the capacity to undertake a similar, and expanding, range of functions is required in any health research system, but is still quite limited in some countries. However, there is a range of initiatives underway to increase health research capacity at system, institution, and individual levels, and many of these are in low- and middle-income countries (LMICs). In June 2014, *Health Research Policy and Systems* (*HARPS*) published a mini-series entitled S*trengthening Institutional Health Systems Research Capacity for Seven Schools of Public Health in East and Central Africa* [2–5]. This series describes how, in a programme funded by the UK’s Department for International Development under the auspices of the Future Health Systems initiative, seven schools of Public Health and selected health policy institutions across six countries in East and Central Africa embarked on a five-year project to strengthen their capacity to undertake high quality, policy-relevant health systems research. The project provides examples of how such activities can continue to be taken forward. For example, Jessani et al. developed a tool for the self-assessment of health systems research capacity which can be used by schools of public health to produce institutional capacity development plans [3].

The increased attention on building health research capacity is also illustrated by the initiative called Enhancing Support for Strengthening the Effectiveness of National Capacity Efforts (ESSENCE on Health Research), a collaborative framework between funding agencies to scale up research capacity and hosted by the Special Programme for Research and Training in Tropical Diseases at the World Health Organisation (WHO). A Canadian/UK team working with ESSENCE on Health Research have published several recent papers in *HARPS*, including ones on indicators for tracking programmes to strengthen health research capacity [6] and on frameworks for evaluating health capacity strengthening [7]. The Canadian and UK members of the team also used peer-reviewed and grey literature to develop a five-step pathway for designing and evaluating health research capacity strengthening programmes, and tested it in a variety of contexts in Africa [8]. Recent research capacity initiatives are not, of course, limited to LMICs. Canadian examples include one led by the Canadian Association for Health Services and Policy Research [9], and another that demonstrated the role networking can play in researchers’ careers and more broadly in research capacity strengthening [10]. The latter did so through examining the opportunity for networking among new global health researchers provided by the annual Summer Institute of the Canadian Coalition for Global Health Research.

Despite progress, the need for the existing and continuing efforts to increase health research capacity are underlined by a recent assessment of publications involving health policy and systems research from the 71 LMICs that had worked with WHO to produce Country Cooperation Strategies. This analysis indicates a continuing need for WHO and other global health agencies to work to build health policy and systems research capacity [11]. A further recent analysis published in *HARPS* shows that even where there has been progress in a country such as India, it can be very patchy and research capacity still needs to be strengthened in many parts of the country [12].

### Using research to inform health policy

As Bacon emphasised, it is also important to give attention to how research can be ‘*of use and practice for man’s life*’ [1]. Almost since its formation in 2003, *HARPS* has had a focus on the role of research in health policymaking, with an early paper [13] building on the work of Kogan and Henkel in the 1970s and 1980s [14]. Kogan and Henkel were pioneers, developing the concept of the collaborative approach between researchers and policymakers as a way of addressing some of the difficulties of promoting research use in policymaking, and also recognising that there is a diversity of situations in which there might potentially be scope for various types of research to have impact on policymaking. *HARPS* subsequently published the influential *SUPPORT Tools for Evidence-Informed Health Policymaking (STP)* series led by Lavis, Oxman, and colleagues [15].

There have been many recent initiatives to strengthen the use of health research for policy, some described in *HARPS*, and the scope of these varies considerably. One example focussed on lessons recently learnt about the knowledge translation platforms (the partnerships between policymakers, stakeholders, and researchers) that are being established in LMICs to enhance evidence-informed health policymaking [16]. In a paper from Australia, but with an international focus, Milat et al. concluded that for ‘scaling-up’ decisions, ‘*Research evidence formed a component of the overall set of information used in decision-making, but its contribution was limited by the paucity of relevant intervention effectiveness research, and data on costs and cost effectiveness.*’ [17]. At a much more specific level, a study from the Netherlands showed how a research contribution mapping approach, developed by Kok and Schuit and first described in *HARPS* [18], could be applied to examine how far a specific project commissioned by the Health Care Inspectorate had contributed to the Inspectorate’s work [19].

The relationship between research and policymaking remains complex, and has still not been fully explored, as Oliver et al. recently described in *HARPS* [20]. In this paper, the authors critically analysed the body of work included in the systematic reviews of studies of the use of evidence by policymakers that were published in 2014 by Oliver et al. [21] and, in 2003, by Innvær et al. [22]. They suggested there should be a greater focus on studying the processes of policymaking, the needs of policymakers, the information they do use, and the diversity of circumstances in which knowledge might be used in policymaking. They also claimed that more needs to be done to evaluate the impact of research on populations.

### Assessing the wider impacts of research

There are, nevertheless, already some approaches that use wider frameworks to explore the impact of research use not only on policymaking but also (sometimes through policymaking) in terms of improved health services, population health, and benefits to the economy. In the UK, the Higher Education Funding Council for England (HEFCE) has developed the Research Excellence Framework [23], in which 20% of the evaluation of all university research will depend on the assessment of the wider impact made by the research on non-academic audiences. In a memorandum to the UK Parliament, HEFCE described how it had developed proposals to assess the wider impact, and in doing so explained that the evidence it drew on came from methods to assess the impact of health research. HEFCE stated: ‘*In developing our proposal we drew heavily on the existing evidence. There have been a number of studies that have estimated the impact of research*’. The two examples provided to support this claim were: ‘*work using the “payback framework” …* [and] *Medical Research: What’s it Worth … these studies have helped us to identify common methodological challenges and have shown how they can be overcome.*’ [23].

Building on the same body of evidence, the World Health Report 2013: *Health Research for Universal Coverage* stated: ‘*adding impetus to do more research is a growing body of evidence on the returns on investments … there is mounting quantitative proof of the benefits of research to health, society and the economy.*’ [24]. In support of this statement, they cited the UK study, *Medical Research: What’s it Worth* [25]. Further analysis also drew on studies from the US and Australia [26–28]. However, the World Health Report also claimed that ‘*Not all the benefits of research can be, or should be, measured in monetary terms. To capture the diversity of benefits from research, the Payback Framework evaluates outcomes under five headings: knowledge, benefits to future research and research use, benefits from informing policy and product development, health and health sector benefits, and economic benefits.*’ [24].

While the first account of the Payback Framework was in 1996 [29], a major update was described in *HARPS* in 2004 [30]. Two reviews of studies assessing the wider impacts from health research have been published in *HARPS*, one from the Italian Cochrane Centre [31] and another from an Iranian team [32], both identifying the Payback Framework as the most widely used approach, including its influence on other frameworks. Papers published in *HARPS* that describe applications and developments of the Payback Framework include two on primary care research in Australia [33, 34].

There have also been important developments in the assessment of the impact of health research in North America. A panel of the Canadian Academy of Health Sciences (CAHS) built on the Payback Framework to recommend ‘*A preferred framework and indicators to measure returns on investment in health research*’ [35]. This was viewed as a ‘*much awaited development in the Canadian health research community*’ [36] and is being developed and applied by various research funders in Canada, for example by *Alberta Innovates – Health Solutions* [36]. The CAHS development of the Payback Framework has also been applied in Spain. An article in *HARPS* describing this application drew on interviews that demonstrated how projects on respiratory diseases funded by the Agency for Health Quality and Assessment of Catalonia between 1996 and 2004, ‘*indicated changes in health services or clinical practice had resulted from research.*’ [37]. There has, of course, been interest in these issues for many years, as traced by a paper considering some of the history of medical research in the USA [38]. In keeping with the growing interest, the Scientific Management Board of the National Institutes of Health (NIH) produced a report in 2014 exploring the current approaches to assessing the value of biomedical research, and recommended the establishment of a trans-NIH committee to develop a strategy and take forward work in this field [39].

Similarly, there are many developments in Europe, including in the Netherlands where an example from Leiden University Medical Center was reported in a paper in *HARPS* [40]. As noted above, the UK has probably seen most activity, and in addition to the approaches previously described, there have also been attempts to develop a performance monitoring framework for the English National Institute for Health Research [41].

Perhaps crucially in terms of taking the field forward, 2014 looks as though it could be an important year in demonstrating the impact of health research; this will be in several ways. First, there have recently been some key developments in the stream of work assessing the value of medical research. The original 2008 study in the UK, *Medical Research: What’s it Worth* [25], showed that on average every pound of public and charitable funding spent on cardiovascular and mental health research generated a very much higher level of benefits than the standard minimum required by the UK Treasury for the investment of public money. In June 2014, the same team published a follow-up study examining the value of UK cancer research and found that the best estimate for the rate of returns is even higher than in the previous study [42]. The particular significance of this 2014 study is that, given that cancer, cardiovascular disease, and mental health disorders account for approximately 45% of the current burden of disease in the UK, it can be concluded that investments in medical research produce a sizeable return in areas where there is a high morbidity [42].

A second factor making 2014 a pivotal year in this field is that December will see the results of the assessment of the 7,000 impact case studies submitted to the UK exercise to evaluate the quality and impact of university research. While this exercise covers all university researchers, well over 1,500 of the case studies will demonstrate the wider impacts of health and biomedical research from UK universities, including medical schools. The wider impacts eligible for consideration in the evaluation include impacts in the following categories: informing health policies and product development; the behaviour of practitioners and members of the public; improved health and health systems; and economic benefits. The database of case studies of the impact of health research will facilitate analysis of features of the full health research system in the UK that might have contributed to the impact. This, too, should help to demonstrate the value of the impacts made by funding health research, an increasingly important issue in many countries.

### Building health research systems to meet the needs of health systems

The editors of *HARPS* believe it is useful to adopt a systems approach when considering how best to develop health research in all countries [43], and are pleased that such an approach was promoted in the 2013 World Health Report [24]. *HARPS* has published the findings from multi-country surveys of progress in developing health research systems, including in Latin American and Caribbean countries [44], the Economic Community of the West African States [45], and the 27 countries of the European Union [46]. Country-specific papers include ones on Panama [47], Guinea Bissau [48], Zambia [49], Solomon Islands [50], and England [51]. Contrasting systems at different stages of development have now been analysed and highlight a number of common key issues that are sometimes already being addressed, and often still need much more action. Such issues include research agenda setting, capacity building, and enhancing the use of research findings.

The importance of action at the systems level is increasingly becoming clear. In relation to agenda-setting, the involvement of a range of stakeholders is seen as important. *HARPS* recently published a paper that followed on from the overall analysis of the Panamanian health research system described above [52]; the follow-on paper examined previous attempts to set priorities for health research in Panama. To inform the approach taken, the analysis partly drew on an earlier paper in *HARPS* by Viergever et al. [53]. We also published a paper in 2014 in which Kothari et al. described the success of a process of consensus building between diverse stakeholders to build a public health systems research agenda in Ontario, Canada, using an approach developed in the USA [54]. *HARPS* is also interested in helping to promote research approaches that are relevant for addressing key issues. Hence, we have just published a major new article collection that was organised by Adam and colleagues at the Alliance for Health Policy and Systems Research: *Advancing the Application of Systems Thinking in Health*. It includes a commentary that sets out the benefits of using systems thinking in health [55], and an editorial outlining the full range of articles in the collection [56].

We have seen that *HARPS* is increasingly becoming a clearinghouse of challenges and solutions to strengthen health research systems. We intend that *HARPS* will continue to demonstrate how research systems can contribute to addressing key issues facing health systems. For example, the contribution of research systems to addressing existing Millennium Development Goals, and future ones, could become increasingly important as analysis of progress in achieving existing ones intensifies [57]. We hope to feature a future series of papers on a research programme related to this theme, in addition to papers on issues facing the health research systems such as how to analyse and reduce the time taken between the conduct of research and its impact on improving healthcare.

Of course, the system Bacon set out in the utopian vision of *New Atlantis* had many differences from modern systems, but some of the aspirations he set out provide a useful context to examine the progress being made. Health research systems are now being developed in many countries, and the impacts from health research, including on health systems, are now being more systematically assessed. At *HARPS* we shall aim to continue providing a platform for a wide range of contributions on these topics. In the words of the World Health Report, 2013: ‘*All nations will benefit from taking a systematic approach to the monitoring and evaluation of research investments, practices, outputs and applications.*’ [24].

## Abbreviations

CAHS: Canadian Academy of Health Sciences
ESSENCE on Health Research: Enhancing Support for Strengthening the Effectiveness of National Capacity Efforts
HARPS: Health Research Policy and Systems
HEFCE: Higher Education Funding Council for England
LMICs: Low- and middle-income countries
NIH: National Institutes of Health
WHO: World Health Organisation.
